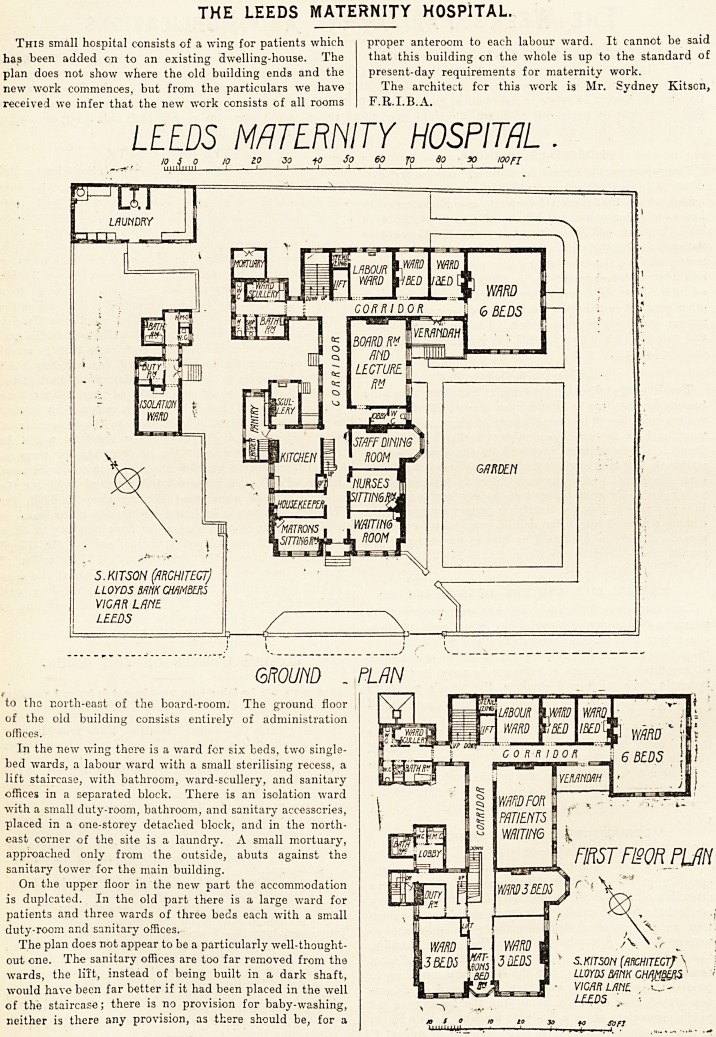# The Leeds Maternity Hospital

**Published:** 1910-07-09

**Authors:** 


					446 THE HOSPITAL. July 9, 1910.
THE LEEDS MATERNITY HOSPITAL.
This small hospital consists of a wing for patients which
has been added on to an existing dwelling-house. The
plan does not show where the old building ends and the
new work commences, but from the particulars we have
received we infer that the new work consists of all rooms
to the north-east of the board-room. The ground floor
of the old building consists entirely of administration
offices.
In the new wing there is a ward for six beds, two single-
bed wards, a labour ward with a small sterilising recess, a
lift staircase, with bathroom, ward-scullery, and sanitary
offices in a separated block. There is an isolation ward
with a small duty-room, bathroom, and sanitary accessories,
placed in a one-storey detached block, and in the north-
east corner of the site is a laundry. A small mortuary,
approached only from the outside, abuts against the
sanitary tower for the main building.
On the upper floor in the new part the accommodation
is duplcated. In the old part there is a large ward for
patients and three wards of three beds each with a small
duty-room and sanitary offices.
The plan does not appear to be a particularly well-thought-
out one. The sanitary offices are too far removed from the
wards, the lilt, instead of being built in a dark shaft,
would have been far better if it had been placed in the well
of the staircase; there is no provision for baby-washing,
neither is there any provision, as there should be, for a
proper anteroom to each labour ward. It cannot be said
that this building on the whole is up to the standard of
present-day requirements for maternity work.
The architect for this work is Mr. Sydney Kitson,
F.R.I.B.A.
THE LEEDS MATERNITY HOSPITAL.
This small hospital consists of a wing for patients which
has been added on to an existing dwelling-house. The
plan does not show where the old building ends and the
new work commences, but from the particulars we have
received we infer that the new work consists of all rooms
proper anteroom to each labour ward. It cannot be said
that this building on the whole is up to the standard of
present-day requirements for maternity work.
The architect for this work is Mr. Sydney Kitson,
F.R.I.B.A.
LEEDS MATERNITY HOSPITAL
10 S 0 to 10 JO +0 So 60 JO do 30 IOOFI
. milium 1 .?i 1 .?i 1 1 1 1 ?i 1
ground _ plan
to the north-east of the board-room. The ground floor
of the old building consists entirely of administration
offices.
In the new wing there is a ward for six beds, two single-
bed wards, a labour ward with a small sterilising recess, a
lift staircase, with bathroom, ward-scullery, and sanitary
offices in a separated block. There is an isolation ward
with a small duty-room, bathroom, and sanitary accessories,
placed in a one-storey detached block, and in the north-
east corner of the site is a laundry. A small mortuary,
approached only from the outside, abuts against the
sanitary tower for the main building.
On the upper floor in the new part the accommodation
is duplcated. In the old part there is a large ward for
patients and three wards of three beds each with a small
duty-room and sanitary offices.
The plan does not appear to be a particularly well-thought-
out one. The sanitary offices are too far removed from the
wards, the lift, instead of being built in a dark shaft,
would have been far better if it had been placed in the well
of the staircase; there is no provision for baby-washing,
neither is there any provision, as there should be, for a
wwm
PATIENTS
WAITING | I
first Fm nm
S. KITSON [ARCHITECTJ \
LLOYDS Mm CHAMBERS
vicm Lfmt C-
LEZD5 ' '?

				

## Figures and Tables

**Figure f1:**